# Aerodynamic Analysis of Cup Anemometers Performance: The Stationary Harmonic Response

**DOI:** 10.1155/2013/197325

**Published:** 2013-12-04

**Authors:** Santiago Pindado, Javier Cubas, Ángel Sanz-Andrés

**Affiliations:** Universidad Politécnica de Madrid, ETSI Aeronáuticos, Instituto Universitario de Microgravedad “Ignacio Da Riva” (IDR/UPM), Plaza del Cardenal Cisneros 3, 28040 Madrid, Spain

## Abstract

The effect of cup anemometer shape parameters, such as the cups' shape, their size, and their center rotation radius, was experimentally analyzed. This analysis was based on both the calibration constants of the transfer function and the most important harmonic term of the rotor's movement, which due to the cup anemometer design is the third one. This harmonic analysis represents a new approach to study cup anemometer performances. The results clearly showed a good correlation between the average rotational speed of the anemometer's rotor and the mentioned third harmonic term of its movement.

## 1. Introduction

The importance of wind energy for modern societies is today a fact [1]. Some countries have clearly supported this industry at the end of the twentieth century (Denmark, Germany, Spain…), and as a result they have led most of the technical advances on the scientific areas related to wind energy. On the other hand, some other countries have started to invest more and more in their wind energy sector, the consequence being that at present these new players not only show the highest figures of wind energy installed power but also the highest growing rates (China, USA, and India); see Table 1.

As the extractable wind power is proportional to the third power of the wind speed [2], the wind industry demands the best instruments to measure it, with special interest in two particular aspects: wind energy forecast on the field and wind turbine performance control [3]. This fact has made the wind energy sector a mass consumer of cup anemometers all over the world.

Despite technological advances as LIDAR or SODAR [4–7], the cup anemometer, invented in the XIX century for meteorological purposes [8], remains today the most proper instrument for the mentioned tasks of wind energy forecast on the field and wind turbine control. Furthermore, following the IEC-61400-12-1 standard [9], the power performance of a wind turbine is preferred to be based on the wind speed measurements performed with a calibrated cup anemometer [10, 11]. Taking into account the great importance of the wind speed measurements accuracy, a huge work was done during the XX century by researchers and scientists to improve the cup anemometer and to have a better understanding of its performance. After initial efforts to study and optimize the size of the anemometer [12–14], the cup aerodynamics [15, 16], and the output frequency recording systems [17–21], the researchers focused on the analytical and experimental analysis of anemometer performance in the field [16, 19, 22, 23]. Obviously, the aforementioned studies were possible thanks to the advances in experimental techniques achieved in the first part of the XX century.

From the point of view of the wind energy industry, the cup anemometer performance is based on the transfer function:

(1) $$\begin{array}{c} V=A\cdot f+B, \end{array}$$

where *V* is the wind speed, *f* is the anemometer's rotation frequency output, and *A* (slope) and *B* (offset) are the calibration coefficients. This linear equation, which correlates the wind speed and the anemometer's output frequency [24], must be defined by means of a calibration process [9, 25–27] and was quite early defined for the Robinson-type anemometer [28]. The transfer function can be rewritten in terms of the anemometer's rotation frequency, *f*_*r*_, instead of the output frequency, *f*, introducing in the expression the number of pulses per revolution given by the anemometer, *N*_*p*_:

(2) $$\begin{array}{c} V=A\cdot N_{p}\cdot f_{r}+B=A_{r}\cdot f_{r}+B. \end{array}$$

The expression above is preferable than the preceding one, as it has a clearer physical meaning. Expression (2) also allows a direct comparison between anemometers [29] and between experimental results and analytical models [30, 31]. The number of pulses, *N*_*p*_, is different depending on the anemometer's inner system for translating the rotation into electric pulses. Magnet-based systems give 1 to 3 pulses per revolution, whereas optoelectronics-based systems normally give higher pulse rates per revolution, from 6 to 44 [29].

Leaving aside the wide acceptance of the industry, it is also fair to recognize the existence of some special uncertainties associated with the cup anemometer wind speed measurements. On the one hand we have the “overspeeding” problem, which was detected and studied from the beginning of the XX century [32, 33]. The cup anemometer “overspeeding” consists in a quicker response upon wind flow acceleration than the one obtained after a wind flow deceleration. Due to the impact on the measured wind speed and turbulence, this effect (together with the problems related to the error caused by the vertical component of the wind) was one of the biggest concerns for meteorologists during the second half of the XX century [34–41]. All the researches done were quickly applied to the wind energy industry, particularly to the effect of the accuracy on the wind turbine power [42], and the classification and improvement of cup anemometers [43].

From 2009, cup anemometer performances have been analyzed at the IDR/UPM calibration lab, focusing on the effect of the rotor shape [29, 44], the effect of air density and climatic conditions [45], and the effect of aging [46]. Recently, research done at the IDR/UPM has been focused on the uniformity of anemometer rotation, as even in a very low turbulence and stationary wind flow the rotation speed is not purely constant, being composed by harmonic terms.

The cup anemometer has a standardized configuration of three cups, as the 3-cup anemometer has become the most efficient solution when compared to the 4-cup anemometer (this was the initial configuration of the cup anemometer developed in the XIX century). This 3-cup design makes the rotational speed of this instrument not uniform [47]. The rotational speed of a 3-cup anemometer, *ω*, under a perfectly constant and uniform wind speed can be decomposed along one turn into a constant term, *ω*_0_, plus a series of harmonic terms that correspond to a frequency three times bigger than the one related to the mentioned constant term, 3*ω*_0_, and its multiples, 6*ω*_0_, 9*ω*_0_, 12*ω*_0_…:

(3) $$\begin{array}{c} \omega \left(t\right)=\omega_{0}+\overset{\infty }{\underset{n=1}{\sum }}\omega_{3n}\sin\left(3n\omega_{0}t+\varphi_{3n}\right). \end{array}$$

**Figure 1:**
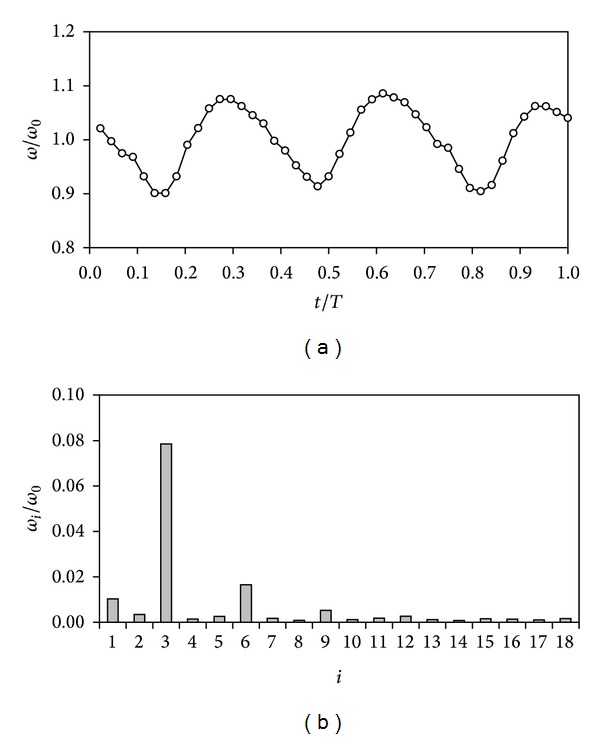
Relative-to-the-average rotational speed, *ω*/*ω*_0_, of a Thies 4.3303 anemometer during one turn at 8 m/s wind speed [47] (a) and non-dimensional values of the Fourier series decomposition performed on that rotational speed, *ω*_*i*_/*ω*_0_ (b). *T* is the period of the anemometer's rotation.

In Figure 1, the non-dimensional rotation speed, *ω*(*t*)/*ω*_0_, of a Thies 4.3303 anemometer at 8 m/s wind flow is shown. The third harmonic term can be clearly appreciated as the most important of the rotation speed, obviously leaving aside the constant term, *ω*_0_. The aforementioned study on the regularity of the rotation speed of the anemometers at constant wind speed revealed the effect of the anemometer's rotor shape on this third harmonic term of the rotation speed (these results were presented at the Alternative Energies Special Session of the 9th Conference on Diffusion in Solids and Liquids; Madrid, 2013). Nevertheless, the results were not conclusive enough, as the analysis was performed using an Ornytion 107A anemometer. This anemometer gives an analog output consisting in 2-harmonic pulses per turn generated by rotating magnets, which is a good enough rate to measure correctly the average rotation speed, *ω*_0_. However, the process to extract the third harmonic term from the output had to be indirect and derived from Lenz's law, which relates the generated analog output of the anemometer to the rotating magnetic field.

The aim of the present study is to analyze the response of an optoelectronic output anemometer (Climatronics 100075 by Climatronics Corp., also known as F460 model), equipped with different rotors, to have a better understanding of the effect of the geometry (size of the cups, distance of the cups to the rotation axis) on cup anemometer performances. Also, the third harmonic term of the rotation speed, see expression (3), is studied as a possible new approach to analyze the mentioned anemometer performances.

## 2. Testing Configuration and Cases Studied

**Figure 2:**
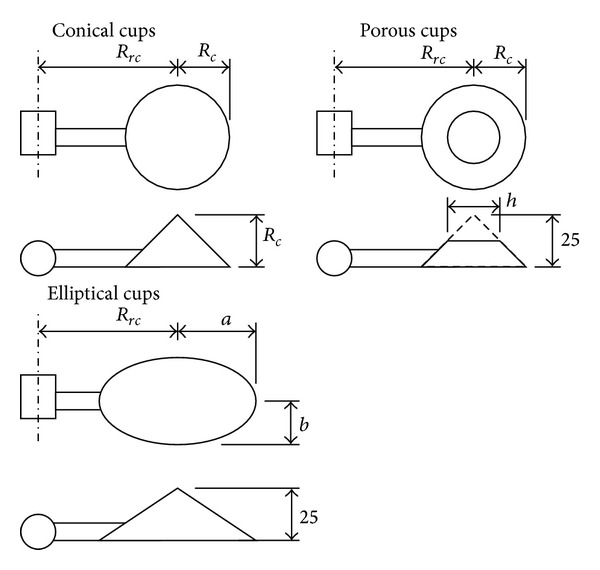
Sketch of the cups and rotor geometries tested. Dimensions in mm. See also Table 2.

As said, the Climatronics 100075 anemometer was used in the testing campaign. 32 different rotors were tested (see Table 2 and Figure 2): 26 were equipped with conical cups (90° cone-angle, 4 with cup radius: *R*_*c*_ = 20 mm, cup center rotation radius varying from *R*_*rc*_ = 30 mm to *R*_*rc*_ = 60 mm; 5 with cup radius: *R*_*c*_ = 25 mm, cup center rotation radius varying from *R*_*rc*_ = 40 mm to *R*_*rc*_ = 100 mm; 6 with cup radius: *R*_*c*_ = 30 mm, cup center rotation radius varying from *R*_*rc*_ = 40 mm to *R*_*rc*_ = 120 mm; 5 with cup radius: *R*_*c*_ = 35 mm, cup center rotation radius varying from *R*_*rc*_ = 50 mm to *R*_*rc*_ = 120 mm; and 6 with cup radius: *R*_*c*_ = 40 mm, cup center rotation radius varying from *R*_*rc*_ = 60 mm to *R*_*rc*_ = 140 mm). 3 were equipped with elliptical cups (front surface equal to the conical cups: *S*_*c*_ = 1963.5 mm^2^ and *R*_*rc*_ = 60 mm). 3 were equipped with porous cups (front surface, including the empty area, equal to the conical cups: *S*_*c*_ = 1963.5 mm^2^, cup radius: *R*_*c*_ = 25 mm, truncated shape with hole diameter *h* = 9 mm, *h* = 19 mm and *h* = 24 mm, and *R*_*rc*_ = 60 mm). The cups used in this study were made of ABS plastic using a 3D printer, and the arm on each cup was made of aluminum tubing 5 mm in diameter.

**Figure 3:**
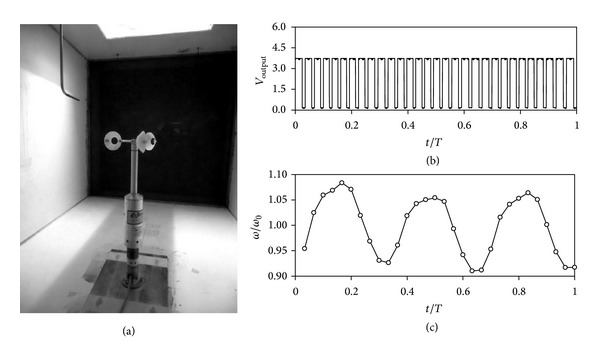
Climatronics 100075 anemometer placed in the S4 wind tunnel of the IDR/UPM Institute to carry out a calibration with the h-19/60 rotor (a). Anemometer voltage output signal, *V*_output_, in one turn (b). Relative-to-the-average rotational speed, *ω*/*ω*_0_, in one turn (c). *T* is the period of the anemometer's rotation.

The calibrations were carried out at the IDR/UPM Institute, in the S4 wind tunnel (see Figure 3). This facility is an open-circuit wind tunnel with a closed test section measuring 0.9 by 0.9 m. It is served by four 7.5 kW fans with a flow uniformity under 0.2% in the testing area. More details concerning the facility and the calibration process are included in [29]. The calibrations analyzed in the present paper were performed following the MEASNET [25, 26] recommendations (over 13 points and from 4 to 16 m/s wind speed). In each point (wind speed) of every calibration performed, the anemometer's output was sampled during 20 seconds at 10000 Hz.

The Climatronics 100075 anemometer gives 30 squared pulses per turn. A plot of the output signal record in one turn of this anemometer equipped with the h-24/60 rotor is included in Figure 3, together with the non-dimensional rotation speed, *ω*/*ω*_0_, once the output was post-processed. The non-dimensional rotation speed on one turn was calculated averaging groups of 30 pulses contained in the recorded data for every point (i.e., every wind flow velocity), during the calibration of all configurations analyzed.

## 3. Results and Discussion

**Figure 4:**
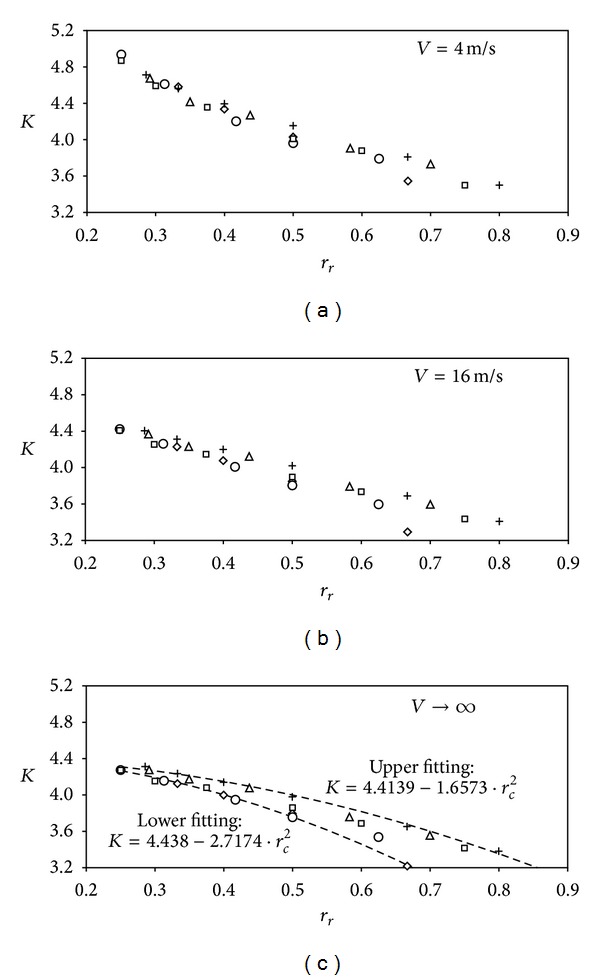
Anemometer factor, *K*, regarding the conical cups rotors, calculated for *V* = 4 m/s (a), *V* = 16 m/s (b), and *V* → *∞* (c), as a function of the parameter *r*_*r*_ (*r*_*r*_ = *R*_*c*_/*R*_*rc*_). The symbols correspond to rotors equipped with the following cup radii: *R*_*c*_ = 20 mm (rhombi), *R*_*c*_ = 25 mm (circles), *R*_*c*_ = 30 mm (squares), *R*_*c*_ = 35 mm (triangles), and *R*_*c*_ = 40 mm (crosses). The quadratic fittings to *R*_*c*_ = 20 mm rotors (lower fitting) and to *R*_*c*_ = 40 mm rotors (upper fitting) have been also added (dotted lines) to the bottom graph.

The calibration constants of the anemometer transfer function, *A* and *B*, with regard to all cases tested are included in Table 3. In Figure 4 the anemometer factor, *K*, regarding the calibrations performed in the present study to conical cups rotors is shown as a function of the parameter *r*_*r*_ (which represents the ratio between the cups' radius, *R*_*c*_, and the cups' center rotation radius, *R*_*rc*_, *r*_*r*_ = *R*_*c*_/*R*_*rc*_). This anemometer factor is defined as the ratio between the wind speed, *V*, and the rotation speed of the cup center averaged in one complete rotation, *ω*_0_ *R*_*rc*_ [48]:

(4) $$\begin{array}{c} K=\frac{V}{\omega_{0}R_{rc}}=\frac{A_{r}f_{r}+B}{2\pi f_{r}R_{rc}}=\frac{A_{r}}{2\pi R_{rc}}\frac{1}{1-\left(B/V\right)}, \end{array}$$

where *A*_*r*_ and *B* are, respectively, the slope and the offset of the transfer function; see expression (2). The anemometer factor, *K*, was calculated for both limits of the wind speed calibration range, 4 m/s and 16 m/s, and for very high wind speeds (*V* → *∞*), which, in fact, is the result of considering the offset constant of the transfer function, *B*, negligible. It should be said that the effect of this offset constant on similar calculations has been considered negligible in past works [44, 48], as it is only appreciated at low wind speeds. However, it was preserved in the present calculations in order to have a more accurate comparison between the different cases, and new conclusions have arisen.

In Figure 4 three different cases can be observed. For low wind speeds (*V* = 4 m/s) the cup anemometer is less efficient in terms of transforming the wind velocity into rotational speed than for higher wind speeds (i.e., higher values of the anemometer factor, *K*, are shown). Also, the curves corresponding to the different cup sizes seem to follow the same path for ratios between the cups' radius and the cups' center rotation radius lower than *r*_*r*_ < 0.65. The mentioned lower performances of the anemometer can be explained as an effect of the friction forces, which are increasingly significant when compared to the aerodynamic forces for low wind speeds (that, obviously, are translated into low rotational speeds).

The situation changes for higher wind speeds, as it can be observed for *V* = 16 m/s and even more clearly for the limit case *V* → *∞* (i.e., when the offset constant *B* is left aside). In this case, the anemometer constant, *K*, shows a second-order polynomial dependence on the parameter *r*_*r*_. Also, the effect of relative cup size is shown in the mentioned graph. The curves fitting to the results corresponding to the rotors with the smallest and the largest cups (*R*_*c*_ = 20 mm and *R*_*c*_ = 40 mm, resp.) have been included in the graph. The results corresponding to all the intermediate cup size rotors (*R*_*c*_ = 25 mm, *R*_*c*_ = 30 mm, and *R*_*c*_ = 35 mm) lie between both curves revealing the aforementioned dependence on the cups' size. In tune with this effect, it should also be said that other experimental results have already demonstrated the direct relationship between the slope of the anemometer transfer function, *A*, and the front area of the cups [44]:

(5) $$\begin{array}{c} A=\frac{1}{N_{p}}A_{r}=\frac{1}{N_{p}}\left(\frac{\text{d}A_{r}}{\text{d}R_{rc}}R_{rc}+A_{r0}\right), \end{array}$$

where d*A*_*r*_/d*R*_*rc*_ depends on the aerodynamic forces on the cups (for rotors equipped with the same conical cups tested in the present work, it was found that this coefficient has constant value with very little or no correlation to the cups' size) and *A*_*r*0_ strongly depends on the cups' front area, *S*_*c*_. Finally, it should also be said that as far as the authors know, this particular effect of the cups' size has not been included in the different analytical models developed to study cup anemometer behavior [30, 31, 33, 38, 40]. These models are based on wind speed, cup aerodynamic coefficients, and cup and rotor geometries and take as starting point that the behavior of cup anemometers is mainly driven by aerodynamic forces, the frictional torque being much lower in comparison [37, 49]. However, these models are limited due to the complexity of rotating flows [48].

In order go to deeper into this problem, the aerodynamic forces on each cup should be analyzed. As the aerodynamic torque on the anemometer's rotor is produced by the aerodynamic forces on the mentioned cups and the cups positions on the rotor have 120° phase separation, it is logical to assume that at constant wind speed equal rotational accelerations and decelerations will affect the anemometer rotor three times per revolution (obviously, these accelerations are responsible for the third harmonic term of the anemometer wind speed, *ω*_3_; see expression (3)). Therefore, studying the third harmonic term of the rotational speed is a way to analyze the effect of the cups on the rotor movement.

**Figure 5:**
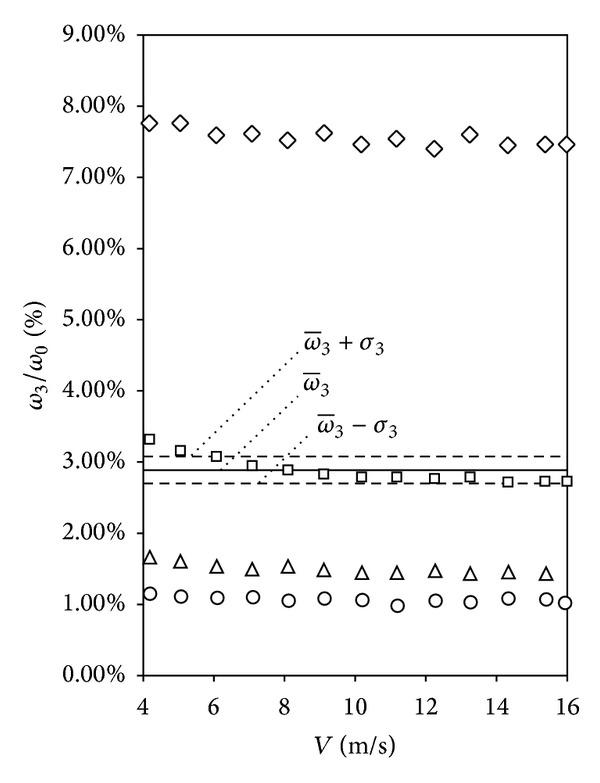
Non-dimensional harmonic term, *ω*_3_/*ω*_0_, calculated at every point of the calibrations performed on the Climatronics 100075 anemometer equipped with c-25/60 (circles), h-09/60 (triangles), h-19/60 (squares), and h-24/60 (rhombi) rotors. The average value line, ${\overset{-}{\omega }}_{3}$, together with the standard deviation limits, ±*σ*_3_, corresponding to the h-19/60 rotor values has been also included in the graph.

In Figure 5, the non-dimensional third harmonic term, *ω*_3_/*ω*_0_, calculated for every wind speed of the calibrations regarding the studied porous cups rotors (h-09/60, h-19/60 and h-24/60), is shown together with the results corresponding to the c-25/60 rotor (included as it can be considered as part of the porous cup series; i.e., porosity equal to zero). In the mentioned figure the differences among the performances with regard to the different rotors can be clearly observed. This figure has been chosen to illustrate two parameters used in the present work to analyze the third harmonic term of the anemometer rotational speed: (i) the non-dimensional average value calculated with data from the 13 points of the calibration procedure:

(6) $$\begin{array}{c} {\overset{-}{\omega }}_{3}=\frac{1}{13}{\overset{13}{\underset{i=1}{\sum }}\frac{\omega_{3}}{\omega_{0}}|}_{i}, \end{array}$$

and (ii) the corresponding standard deviation, *σ*_3_, calculated using the general procedure:

(7) $$\begin{array}{c} \sigma_{3}=\sqrt{\frac{\sum_{i=1}^{13}{\left({\left(\omega_{3}/\omega_{0}\right)|}_{i}-{\overset{-}{\omega }}_{3}\right)}^{2}}{13-1}}. \end{array}$$

**Figure 6:**
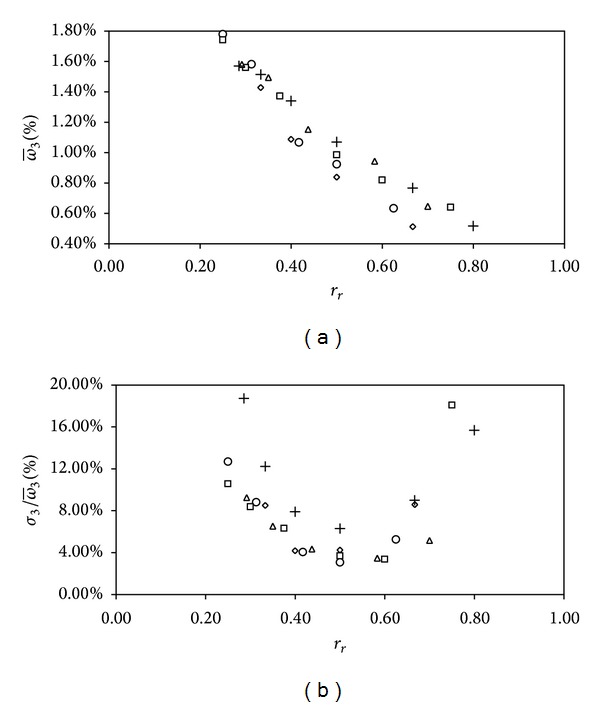
Averaged third harmonic term, ${\overset{-}{\omega }}_{3}$, regarding the conical cups rotors (a), and the corresponding standard deviation, $\sigma_{3}/{\overset{-}{\omega }}_{3}$ (b), both expressed as a function of the parameter *r*_*r*_ (*r*_*r*_ = *R*_*c*_/*R*_*rc*_). The symbols correspond to rotors equipped with the following cup radii: *R*_*c*_ = 20 mm (rhombi), *R*_*c*_ = 25 mm (circles), *R*_*c*_ = 30 mm (squares), *R*_*c*_ = 35 mm (triangles), and *R*_*c*_ = 40 mm (crosses).

In Figure 6 the averaged third non-dimensional harmonic term, ${\overset{-}{\omega }}_{3}$, regarding the conical cups rotors, and the ratio of the standard deviation to the mentioned non-dimensional third harmonic term, $\sigma_{3}/{\overset{-}{\omega }}_{3}$, are shown as a function of the ratio of the cup radius to the cups' center rotation radius, *r*_*r*_. The third harmonic term, ${\overset{-}{\omega }}_{3}$, tends to be smaller with higher values of *r*_*r*_, that is, for rotors whose cups centers are closer to the rotation axis. The same tendency was observed on the anemometer factor, *K* (see Figure 4), so it can be concluded that higher third harmonic terms have an immediate effect on the anemometer average performance, reducing the average rotational speed, *ω*_0_. This effect can be explained in terms of energy, as a bigger part of the energy transferred from the wind to the rotor movement is invested into the mentioned third harmonic term and not into the constant term of the rotational speed, *ω*_0_. Also, the effect of the cups' size has the same pattern as the one for the anemometer constant graphs in Figure 4. Smaller cups with the same parameter *r*_*r*_ show smaller third harmonic terms, with higher rotational efficiencies.

**Figure 7:**
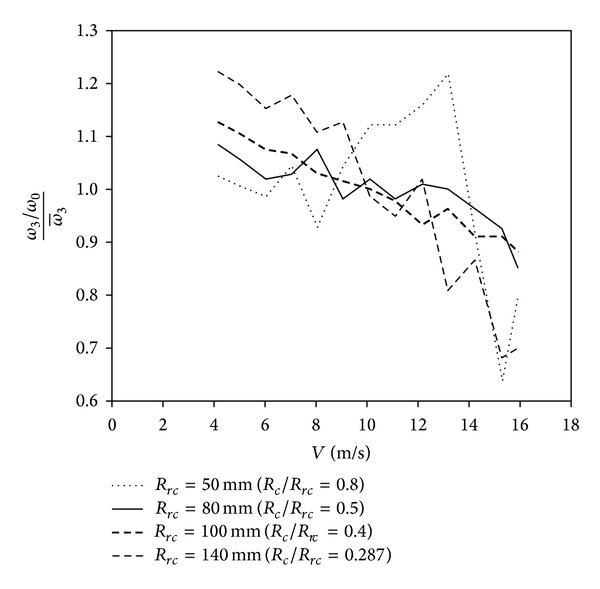
Variation of the third harmonic term measured with rotors equipped with *R*_*c*_ = 40 mm conical cups as a function of the wind speed within the calibration process.

Finally, focusing on the standard deviation measured with respect to the average third harmonic term, $\sigma_{3}/{\overset{-}{\omega }}_{3}$, shown in Figure 6, it seems that there is a minimum for every cup size at a certain value of the parameter *r*_*r*_, located around *r*_*r*_ = 0.5. In Figure 7 the third harmonic term measured for every wind velocity during the calibration of four rotors equipped with *R*_*c*_ = 40 mm cups is shown. The data correspond to the smallest and the largest cup rotation radius tested (*R*_*rc*_ = 50 mm and *R*_*rc*_ = 140 mm, resp.), and two intermediate cases (*R*_*rc*_ = 80 mm and *R*_*rc*_ = 100 mm). It can be observed that, according to the information from the mentioned graph in Figure 6, the dispersion of the results is lower for the intermediate cases. A linear tendency is also observed, with higher slopes for greater values of cup center rotation radius. Finally, it should also be said that class-1 commercial anemometers [47, 50] have ratios between the cup radius and the cups' center rotation radius ranging from *r*_*r*_ = 0.4 to *r*_*r*_ = 0.6 (Thies Clima 4.3350/4.3351, *r*_*r*_ = 0.5; Vector Instruments A100L2, *r*_*r*_ = 0.49; Vaisala WAA 151/252, *r*_*r*_ = 0.42; WindSensor (Risø) P2546A, *r*_*r*_ = 0.59).

**Figure 8:**
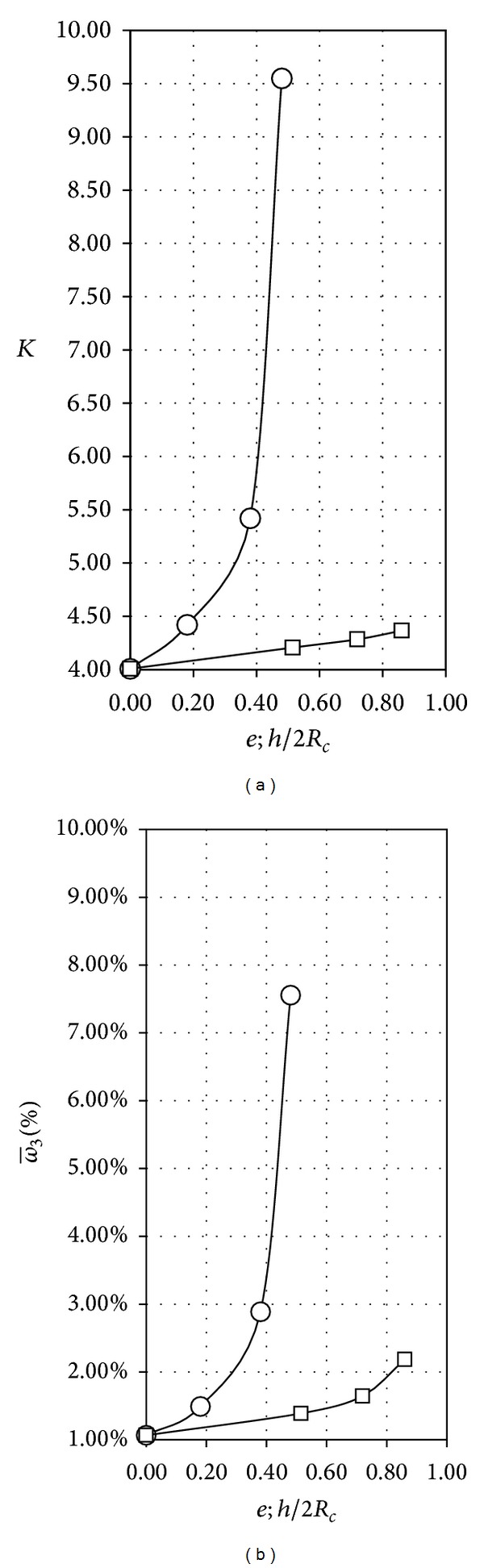
Anemometer constant at 16 m/s wind speed, *K*, and non-dimensional third harmonic, ${\overset{-}{\omega }}_{3}$, with regard to the calibrations performed to the anemometer equipped with elliptical (squares) and porous (circles) cup rotors, as a function of the eccentricity, $e=\sqrt{1-{\left(b/a\right)}^{2}}$, and the ratio of the hole diameter to the front cup area, *h*/2*R*_*c*_, respectively.

Concerning the effect of the cups' shape, its effect on anemometer performance has been already analyzed. The aerodynamic forces on a single cup have been correlated with the aforementioned anemometer performance in two different ways, by means of analytical models correlated with experimental testing [30, 31, 33, 38] and by means of a direct comparison based on experimental calibration [48]. In Figure 8, the anemometer constant at 16 m/s wind speed with regard to the calibrations performed on the anemometer equipped with elliptical and porous cups rotors, has been included, respectively, as a function of the eccentricity, $e=\sqrt{1-{\left(b/a\right)}^{2}}$, and the ratio of the hole diameter to the cup diameter, *h*/2*R*_*c*_. The non-dimensional third harmonic, ${\overset{-}{\omega }}_{3}$, regarding these rotors has been also included in the figure. As it was previously measured [48], the effect of both the eccentricity and the porosity of the cups is translated into a decrease of the rotational efficiency of the cups (higher values of *K*, that is, lower rotational speed at a fixed wind speed). On the other hand, the effect of both parameters on the third harmonic term has a reversed effect; that is, higher values of eccentricity and porosity increase the importance of this harmonic term.

## 4. Conclusions

In the present work, the performance of a cup anemometer equipped with different rotors has been experimentally analyzed. On one hand, the size of conical cups together with their distance to the anemometer rotation axis was studied. On the other hand, the shape of the rotor cups was also studied. The analysis was based on two different parameters, the anemometer factor that takes into account the average rotation of the anemometer and the third harmonic term resulting from the Fourier decomposition of the anemometer rotation speed. The most relevant conclusions resulting from this work are as follows.

- The average rotation speed is well correlated with the mentioned third harmonic term resulting from the Fourier decomposition of the anemometer rotation speed. Higher values of this third harmonic term result in higher anemometer factors and therefore in lower rotational speeds. This fact confirms the mentioned third harmonic term as a useful parameter to study cup anemometer performance.
- Smaller cups with the same ratio of cup diameter to the cups' center rotation radius, *r*_*r*_, tend to produce higher rotational speeds (lower anemometer factors) and lower third harmonic terms.
- The ratio of the third harmonic term to the average rotational speed, *ω*_3_/*ω*_0_, is more uniform (within the wind speed calibration range) for values of the ratio of the cup diameter to the cups' center rotation radius ranging from *r*_*r*_ = 0.4 to *r*_*r*_ = 0.5 than for values of *r*_*r*_ beyond or below this bracket.

## Figures and Tables

**Table 1:** Installed wind power per country (units in GW) of some of the biggest world producers from 2005 to 2012. The growing rates (with respect to the preceding year) corresponding to 2011 and 2012 have been also added in brackets to illustrate the present status of the wind energy market (sources: Global Wind Energy Council; US Energy Information Administration).

Country	2005	2006	2007	2008	2009	2010	2011	2012
China	1.26	2.60	5.91	12.20	16.00	31.10	62.36 (100.5%)	75.56 (21.2%)
USA	8.71	11.33	16.52	24.65	34.30	39.14	46.92 (19.9%)	60.04 (28.0%)
Germany	18.43	20.62	22.25	23.90	25.70	27.20	29.08 (6.9%)	31.51 (8.4%)
Spain	9.92	11.72	14.80	16.60	19.10	20.70	21.67 (4.7%)	22.80 (5.2%)
India	5.30	6.20	7.80	10.00	11.00	13.07	16.08 (23.1%)	18.42 (14.5%)
Italy	1.64	1.90	2.70	3.53	4.88	5.79	6.88 (18.8%)	8.15 (18.5%)
UK	1.57	1.96	2.48	3.41	4.42	5.38	6.47 (20.3%)	8.37 (29.3%)
Canada	0.68	1.46	1.77	2.37	3.32	3.97	5.65 (42.2%)	6.59 (16.5%)
Portugal	1.06	1.68	2.20	2.86	3.33	3.80	4.30 (13.3%)	4.45 (3.4%)
Brazil	0.03	0.24	0.25	0.41	0.60	0.93	1.51 (62.8%)	2.59 (71.4%)

**Table 2:** Geometrical characteristics of the rotors tested: cup center rotation radius, *R*_*rc*_, front area of the cups, *S*_*c*_, cup radius (conical and porous cups), *R*_*c*_, ratio of cup radius to the cups' center rotation radius (conical cups), *R*_*c*_/*R*_*rc*_, hole diameter of porous cups, *h*, and semi-major and semi-minor axes, *a* and *b*, of elliptical cups. See also Figure 2.

Conical cups				
Rotor	*R*_*c*_ [mm]	*S*_*c*_ [mm^2^]	*R*_*rc*_ [mm]	*R*_*c*_/*R*_*rc*_
c-20/30	20	1256.6	30	0.667
c-20/40	20	1256.6	40	0.500
c-20/50	20	1256.6	50	0.400
c-20/60	20	1256.6	60	0.333
c-25/40	25	1963.5	40	0.625
c-25/50	25	1963.5	50	0.500
c-25/60	25	1963.5	60	0.417
c-25/80	25	1963.5	80	0.313
c-25/100	25	1963.5	100	0.250
c-30/40	30	2827.4	40	0.750
c-30/50	30	2827.4	50	0.600
c-30/60	30	2827.4	60	0.500
c-30/80	30	2827.4	80	0.375
c-30/100	30	2827.4	100	0.300
c-30/120	30	2827.4	120	0.250
c-35/50	35	3848.5	50	0.700
c-35/60	35	3848.5	60	0.583
c-35/80	35	3848.5	80	0.438
c-35/100	35	3848.5	100	0.350
c-35/120	35	3848.5	120	0.292
c-40/50	40	5026.5	50	0.800
c-40/60	40	5026.5	60	0.667
c-40/80	40	5026.5	80	0.500
c-40/100	40	5026.5	100	0.400
c-40/120	40	5026.5	120	0.333
c-40/140	40	5026.5	140	0.286

Elliptical cups				
Rotor	*a* [mm]	*b* [mm]	*S*_*c*_ [mm^2^]	*R*_*rc*_ [mm]

a-27/60	27	23.15	1963.5	60
a-30/60	30	20.83	1963.5	60
a-35/60	35	17.86	1963.5	60

Porous cups				
Rotor	*R*_*c*_ [mm]	*S*_*c*_ [mm^2^]	*R*_*rc*_ [mm]	*h* [mm]

h-9/60	25	1963.5	60	9
h-19/60	25	1963.5	60	19
h-24/60	25	1963.5	60	24

**Table 3:** Calibration coefficients, *A* and *B*, measured for the rotors tested with Climatronics 100075 anemometer (see also Table 2 and Figure 2). The coefficient of determination, *R* ^2^, of the curve fittings, and the slope of the transfer function based on the rotation frequency, *A*_*r*_ (see expressions (1) and (2)), have also been included.

Conical cups				
Rotor	*A* [m/pulse]	*A*_*r*_ [m/rev]	*B* [m/s]	*R* ^2^
c-20/30	0.02021	0.60618	0.37096	0.99995
c-20/40	0.03177	0.95318	0.23328	0.99999
c-20/50	0.04186	1.25574	0.31148	0.99999
c-20/60	0.05180	1.55401	0.40091	0.99999
c-25/40	0.02963	0.88886	0.26760	0.99999
c-25/50	0.03932	1.17970	0.20786	0.99999
c-25/60	0.04961	1.48829	0.24245	0.99999
c-25/80	0.06964	2.08928	0.39438	0.99999
c-25/100	0.08952	2.68552	0.53685	0.99999
c-30/40	0.02861	0.85818	0.09551	0.99995
c-30/50	0.03861	1.15844	0.19606	0.99999
c-30/60	0.04850	1.45505	0.14823	0.99998
c-30/80	0.06836	2.05089	0.25403	1.00000
c-30/100	0.08697	2.60900	0.38253	0.99998
c-30/120	0.10738	3.22145	0.49053	0.99999
c-35/50	0.03720	1.11603	0.19081	0.99999
c-35/60	0.04719	1.41582	0.15354	0.99999
c-35/80	0.06827	2.04818	0.18132	0.99998
c-35/100	0.08737	2.62111	0.22168	0.99999
c-35/120	0.10742	3.22259	0.34171	0.99999
c-40/50	0.03539	1.06166	0.13714	0.99999
c-40/60	0.04586	1.37587	0.16652	0.99999
c-40/80	0.06663	1.99876	0.16932	0.99998
c-40/100	0.08668	2.60026	0.23292	0.99996
c-40/120	0.10638	3.19140	0.28805	0.99996
c-40/140	0.12639	3.79164	0.34162	0.99995

Elliptical cups				
Rotor	*A* [m/pulse]	*A*_*r*_ [m/rev]	*B* [m/s]	*R* ^2^

a-27/60	0.05221	1.56617	0.19932	0.99999
a-30/60	0.05306	1.59167	0.23224	0.99998
a-35/60	0.05412	1.62361	0.21593	0.99998

Porous cups				
Rotor	*A* [m/pulse]	*A*_*r*_ [m/rev]	*B* [m/s]	*R* ^2^

h-09/60	0.05445	1.63352	0.31414	0.99999
h-19/60	0.06579	1.97376	0.54539	0.99998
h-24/60	0.11763	3.52881	0.31563	0.99998
